# Vitamin D status in full-term exclusively breastfed infants versus full-term breastfed infants receiving vitamin D supplementation in Thailand: a randomized controlled trial

**DOI:** 10.1186/s12887-021-02849-z

**Published:** 2021-09-01

**Authors:** Chayatat Ruangkit, Sukrit Suwannachat, Pornchanok Wantanakorn, Napapailin Sethaphanich, Surapat Assawawiroonhakarn, Oraporn Dumrongwongsiri

**Affiliations:** 1 Ramathibodi Medical School, Chakri Naruebodindra Medical Institute, Faculty of Medicine Ramathibodi Hospital, Mahidol University, Samut Prakan, Thailand; 2 Chakri Narubodindra Medical Institute, Faculty of Medicine Ramathibodi Hospital, Mahidol University, Samut Prakan, Thailand; 3 Department of Pediatrics, Faculty of Medicine Ramathibodi Hospital, Mahidol University, 270 Rama VI Road, Ratchatewi, Bangkok, 10400 Thailand

**Keywords:** 25OHD, 25-hydroxyvitamin D, Vitamin D deficiency, Vitamin D insufficiency, Vitamin D supplementation, Parathyroid hormone, Exclusively breastfed infants

## Abstract

**Background:**

Many international medical organizations recommend vitamin D supplementation for infants, especially exclusively breastfed infants. In Thailand, however, data regarding the vitamin D status in Thai infants are lacking. Such data would help to support physician decisions and guide medical practice.

**Methods:**

Full-term, exclusively breastfed infants were randomized into two groups at 2 months of age to continue exclusive breastfeeding either without vitamin D supplementation (control group, *n* = 44) or with vitamin D_3_ supplementation at 400 IU/day (intervention group, *n* = 43) until 6 months of age. At 6 months, the serum vitamin D (25OHD) of the infants and their mothers, serum bone marker, and infants’ growth parameters were compared between the two groups.

**Results:**

The infants’ serum 25OHD concentration was lower in the control group than intervention group (20.57 ± 12.66 vs. 46.01 ± 16.42 ng/mL, *p* < 0.01). More infants had vitamin D sufficiency (25OHD of > 20 ng/mL) in the intervention group than control group (93.0% vs. 43.2%, *p* < 0.01). There were no significant differences in the maternal 25OHD concentrations between the control and intervention groups (25.08 ± 7.75 vs. 23.75 ± 7.64 ng/mL, *p* = 0.42). Serum calcium, phosphorus, intact parathyroid hormone, alkaline phosphatase, and infants’ growth parameters were comparable between the two groups. After adjustment for the confounding factors, 25OHD concentration in the intervention group was 25.66 ng/mL higher than the control group (95% confidence interval, 19.07–32.25; *p* < 0.001). Vitamin D supplement contributed to an 88.7% decrease in the prevalence of vitamin D insufficiency/deficiency (relative risk, 0.11; 95% confidence interval, 0.04–0.35; *p* < 0.01).

**Conclusions:**

Most full-term, exclusively breastfed Thai infants have serum vitamin D concentration below sufficiency level at 6 months of age. However, vitamin D supplementation (400 IU/day) improves their vitamin D status and prevents vitamin D deficiency.

**Trial registration:**

The study was pre-registered in the Thai Clinical Trials Registry (TCTR20190622001) on 22/06/2019.

## Background

Vitamin D plays an important role in bone metabolism and affects many extraskeletal organ systems [1]. Severe vitamin D deficiency may cause rickets in infants or children and osteomalacia in adults. The natural production of vitamin D in the skin through sunlight exposure is the primary source of vitamin D in humans. Direct dietary vitamin D intake from natural foods, fortified foods, and supplements is another source of vitamin D for the body. Despite improved nutritional knowledge and medical care, vitamin D deficiency and infantile rickets remain significant global public health challenges in developed and developing countries [2]. Although the vitamin D sufficiency level has not been definitively established, most researchers used a serum total 25-hydroxyvitamin D concentration of 20 ng/mL (50 nmol/L) as a cut-off [2]. In Thailand, for example, depending on their age group and residential location, 24.5–52.2% of children aged 3–13 years had vitamin D levels below the cut-off mentioned above [3].

During the first year of life, breastfeeding is one of the most critical factors to child survival, nutrition, development, and maternal health. The World Health Organization and the United Nations Children’s Fund recommend that infants should be exclusively breastfed for the first 6 months of life [4]. However, breastfed infants are known to be at risk of vitamin D deficiency, especially in areas of high latitude, because the vitamin D content in breast milk can vary depending on the maternal vitamin D status and is often low [5, 6]. Moreover, infants’ exposure to sunlight may be limited because of their geographical location; their parents’ culture, beliefs, or practices; or other reasons. As a result, many international medical organizations recommend vitamin D supplementation for infants, especially those who are exclusively breastfed [7, 8]. For example, AAP guideline in 2008 suggested that vitamin D supplementation should begin in the first few days of life for breastfed and partially breastfed newborns at 400 IU/day and continue until the infant is weaned to at least 1 L or 1 qt of vitamin D–fortified formula or whole milk per day [7]. Despite these international recommendations, adherence to the guidelines is still problematic in many countries [9, 10]. Previous studies have revealed barriers to vitamin D supplementation in infants, including physicians’ beliefs that infants in their geographic area are exposed to adequate sunlight or that breast milk provides sufficient vitamin D, making supplementation unnecessary [11].

In Thailand, routine vitamin D supplementation for exclusively breastfed infants has not been widely practiced. Reasons for this include physicians’ belief that infants’ vitamin D status is adequate without supplementation, the lack of obvious clinical signs of vitamin D deficiency during the exclusive breastfeeding period, and the difficulty in finding suitable vitamin D preparations in Thailand. Most importantly, the local healthcare authorities have not established a national consensus to guide pediatricians on vitamin D supplementation in infants. Currently, data on the vitamin D status among breastfed infants in Thailand are very scarce. There is not enough evidence to establish a guideline to support physician decisions or guide medical practice; therefore, research in this field is urgently needed.

This study was performed to evaluate the effect of vitamin D supplementation on the vitamin D status of exclusively breastfed infants during the first 6 months of life. In addition, the associations among the vitamin D status, serum bone markers, and growth parameters of infants were evaluated.

## Methods

This open-label randomized controlled trial was conducted at Chakri Naruebodindra Medical Institute, Faculty of Medicine Ramathibodi Hospital, Mahidol University, Samut Prakan, Thailand from July 2019 to October 2020. The study protocol was approved by the Ramathibodi Hospital Institutional Review Board (ID 10–61-58). The study was pre-registered in the Thai Clinical Trials Registry (TCTR20190622001) on 22/06/2019.

### Study population and randomization

The study participants were recruited from healthy full-term infants and their mothers who attended the well-baby clinic at Chakri Naruebodindra Medical Institute for a routine 2-month infant checkup and immunization. To be eligible, the infants were required to be 6 to 12 weeks old when they entered the study. Only mothers who performed exclusive breastfeeding (feeding infants only breast milk, ether breast or expressed, without any formula feeding or complementary food) and had an intention to continue exclusive breastfeeding until the infants were 6 months old were approached for their consent to participate in the trial. The exclusion criteria were premature infants with a gestational age of < 37 weeks at birth, infants with a congenital anomaly, introduction of infant formula and/or complementary foods before 6 months of age, and participant withdrawal. After the mothers had provided informed consent, the infants were randomized into two groups: the control group and the intervention group. Randomization was performed using opaque, sealed, sequentially numbered envelopes opened after informed consent. Each envelope contained a computer-generated block of four intervention order randomization assignments. An enrollment log was kept to ensure all envelopes were accounted for and used in the correct order.

### Intervention

Routine health supervision and immunization were provided to both groups of participants under individualized physician discretion. Infants in the intervention group were given vitamin D supplementation (400 IU/day) in the form of a daily 1-mL multivitamin drop (composition per mL: vitamin A, 2000 IU; vitamin B_1_, 2 mg; vitamin B_2_, 2 mg; vitamin B_6_, 1.8 mg; vitamin B_12_, 5 mcg; vitamin C, 40 mg; vitamin D_3_, 400 IU; nicotinamide, 15 mg; dexpanthenol) (Munti-Vim drops; B.L. Hua & Co., Ltd., Bangkok, Thailand). The multivitamin drop was used in the study because there was no commercially available infant vitamin D-only preparation in Thailand at the time of the study. No placebo was given to the infants in the control group. All mothers who participated in the study were instructed to strictly follow the study protocol by feeding infants exclusively with breastmilk until 6 months and adhered to their group assignments. They should contact the study team hot-line if they had any issues, questions, concerns, or when they felt the need to provide anything other than breastmilk to the infant. There was no restriction for the mother regarding their activity or diet. All mothers were informed that they could perform their usual daily routine and consume any food, vitamins, or dietary supplements of their preferences.

At 4 months of age, follow-up appointments were made for all infants to ensure compliance with the study protocol and to refill the vitamin D supplementation in the intervention group. At 6 months of age, follow-up appointments were made for all infants and their mothers to conclude the study and collect blood samples. Both appointments at 4 and 6 months of age were performed in accordance with the infants’ routine checkup and immunization schedule visits.

### Data collection

The infants’ demographic data (place of birth, season at birth, gestational age, and sex) and anthropometric data (weight, length, and head circumference) at birth and at the 2-month visit were collected at the time of study enrollment. At the 4- and 6-month visits, the infants’ anthropometric measurements were repeated. At the 6-month visit, the following laboratory data were obtained for each infant: serum concentrations of vitamin D [25-hydroxyvitamin D_2_ (25OHD_2_), 25-hydroxyvitamin D_3_ (25OHD_3_), and total 25-hydroxyvitamin D (25OHD)], intact parathyroid hormone (iPTH), calcium, phosphorous, and alkaline phosphatase (ALP). Maternal age and serum vitamin D concentrations were also collected at the 6-month visit.

### Biochemical analyses

Serum vitamin D (25OHD_2_ and 25OHD_3_) was analyzed using liquid chromatography-tandem mass spectrometry assays (Agilent 6460 Triple Quad LC/MS equipped with 1290 Infinity LC system; Agilent Technologies, Santa Clara, CA, USA). The limit of quantitation and linearity of 25OHD_2_ and 25OHD_3_ were 3.5–250 ng/mL and 2.5–250 ng/mL, respectively. The results of 25OHD_2_ and 25OHD_3_ were combined and reported as 25OHD (total). Internal quality control was performed before each runs as per manufacturer guidelines. An external quality assessment was performed according to RIQAS (Randox International Quality Assessment Scheme, UK), accredited to ISO 17043:2010. The iPTH concentration was measured using an Elecsys® PTH electrochemiluminescence immunoassay (Cobas e601; Roche Diagnostics, Basel, Switzerland) with a measuring range of 1.20–5000 pg/mL. A sandwich test principle is used in the Elecsys® PTH assay for determining intact PTH, in which a biotinylated monoclonal antibody reacts with the N-terminal fragment (1–37) and a monoclonal antibody tagged with a ruthenium complex reacts with the C-terminal fragment (38–84). The plasma concentrations of calcium, phosphorous, and ALP were measured with the Calcium Gen.2, Phosphate (Inorganic) ver.2, and ALP IFCC Gen.2, respectively, using an automated analyzer (Cobas c501; Roche Diagnostics). Internal quality control runs were performed daily as per manufacturer guidelines. External quality assessment was performed according to the Bio-Rad Laboratories Ltd. EQAS program, accredited to ISO 17043:2010. Vitamin D deficiency was defined as a 25OHD concentration of < 12 ng/mL, insufficiency as 25OHD of 12 to 20 ng/mL, and sufficiency as 25OHD of > 20 ng/mL [8].

### Sample size estimation

A power calculation was used to calculate the sample size needed to evaluate the primary outcome (infants’ 25OHD concentrations). A previous study of 34 full-term, exclusively breastfed infants aged 1 to 6 months (mean age was 81.9 ± 37.6 days) in our institution (Ramathibodi Hospital, Bangkok) showed that the infants’ mean 25OHD concentration was 13.6 ± 7.7 ng/mL (unpublished data). We hypothesized that vitamin D supplementation would increase the 25OHD level by at least 50% in the intervention group or to a mean 25OHD concentration of up to 20.4 ng/mL (sufficiency level) with a standard deviation similar to that in the control group. According to this assumption, 40 infants in each group were required to detect a post-intervention difference in the 25OHD concentration with an alpha of 0.01, 90% power, two-sided. To allow for attrition, an additional 20% or 10 subjects were added to each group. Therefore, 50 was selected as the total number of infants in each group.

### Statistical analysis

A univariate analysis was performed to identify significant differences between the groups. Student’s t-test was used for continuous variables, and the results are presented as mean ± standard deviation. The Mann–Whitney U test was used if the distribution was not normal, and the results are presented as median (interquartile range). Pearson’s chi-square test or Fisher’s exact test was used for categorical variables, and the results are presented as total number (%). Multivariate linear regression analysis, which included the study group (control or intervention), maternal 25OHD concentration, infants’ age, weight at enrollment, sex, and birth season, was used to determine the factors associated with infants’ 25OHD concentration. To demonstrate the differences in serum bone markers between infants with and without vitamin D sufficiency, univariate analysis was applied using an infant serum 25OHD concentration of ≤20 ng/mL as the independent variable. A *p*-value of < 0.05 was considered statistically significant. Stata Statistical Software version 15.1 (StataCorp LLC, College Station, TX, USA) was used for all statistical analyses.

## Results

**Fig. 1:**
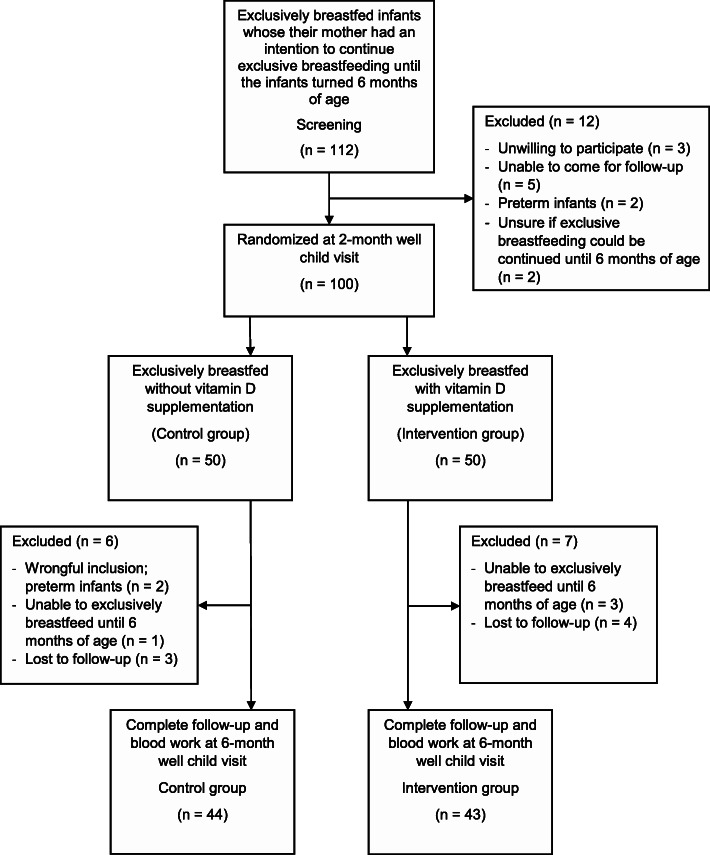
Flow chart of study participants

One hundred mother–infant pairs were randomized to the control and intervention groups according to the study protocol. At the end of the study, 44 and 43 mother–infant pairs in the control and intervention groups, respectively, had completed the study. Figure 1 shows the number of study participants and the reasons for exclusion. The mean age of infants at the time of enrollment and the end of the study were comparable between the control and intervention groups (65 ± 4 vs. 67 ± 6 days, *p* = 0.07 and 184 ± 4 vs. 184 ± 4 days, *p* = 0.86, respectively).

The infants’ weight at birth and enrollment was slightly lower in the control group than in the intervention group. There were no significant differences in the other baseline characteristics of the mothers and infants between the two groups. The demographic data and infants’ birthing season are shown in Table 1.

**Table 1:** Baseline characteristics of study population

Characteristics	Exclusively breastfed without vitamin D supplementation (control group) *n* = 44	Exclusively breastfed with vitamin D supplementation (intervention group) *n* = 43	*p*-value
**Mother**			
Age, years	31.1 ± 4.2	31.3 ± 5.4	0.84
**Infant**			
Sex, male	21 (47.7)	16 (37.2)	0.32
Inborn	26 (59.1)	22 (51.2)	0.46
Gestational age, weeks	38 ± 1	38 ± 1	0.79
Small for gestational age	7 (15.9)	6 (14.0)	0.80
Large for gestational age	1 (2.3)	1 (2.3)	1.00
**Seasons at birth**			0.75
Cool season, Nov–Feb	20 (45.5)	23 (53.5)	
Hot season, Mar–Jun	11 (25.0)	9 (20.9)	
Rainy season, Jul–Oct	13 (29.5)	11 (25.6)	
**At birth**			
Weight, g	3012 ± 373	3176 ± 375	0.05
Length, cm	49.1 ± 1.9	49.4 ± 2.3	0.51
Head circumference, cm	33.5 ± 1.5	33.9 ± 1.3	0.16
**At enrollment**			
Weight, g	4997 ± 423	5302 ± 560	< 0.01
Length, cm	56.6 ± 2.0	57.5 ± 1.9	0.93
Head circumference, cm	38.4 ± 1.2	38.6 ± 1.3	0.38

Data are presented as mean ± standard deviation or n (%)

At four and 6 months follow-up, the infants in the control group still weighed less than those in the intervention group (6251 ± 550 vs. 6588 ± 799 g, respectively, at 4 months, *p* = 0.02, and 7056 ± 694 vs. 7389 ± 837 g, respectively, at 6 months, *p* = 0.04). There were no significant difference in head circumferences between the control and intervention groups during follow-up periods (40.7 ± 1.2 vs. 40.9 ± 1.2 cm, respectively, at 4 months, *p* = 0.39, and 42.3 ± 1.1 vs. 42.7 ± 1.4 cm, respectively, at 6 months, *p* = 0.26) as well no significant difference in the length (62.0 ± 1.9 vs. 62.5 ± 2.0 cm, respectively, at 4 months, *p* = 0.19, and 65.2 ± 2.3 vs. 66.1 ± 2.3 cm, respectively, at 6 months, *p* = 0.06).

At 6 months, the mean 25OHD concentration was lower in the control group than intervention group (20.57 ± 12.66 vs. 46.01 ± 16.42 ng/mL, *p* < 0.01), as shown in Table 2. The prevalences of vitamin D insufficiency and deficiency were lower among infants in the intervention group. Serum bone markers, including calcium, phosphorus, ALP, and iPTH, as well as infants’ growth parameters, were comparable between the two groups. No infants in the study had a clinical manifestation of vitamin D toxicity or clinical rickets.

**Table 2:** Study results

Characteristics	Exclusively breastfed without vitamin D supplementation (control group) *n* = 44	Exclusively breastfed with vitamin D supplementation (intervention group) *n* = 43	*p*-value
**Mother**			
**Vitamin D levels**			
25OHD_2_, ng/mL	0.10 ± 0.38	0.08 ± 0.37	0.83
25OHD_3_, ng/mL	24.97 ± 7.77	23.67 ± 7.70	0.44
25OHD (total), ng/mL	25.08 ± 7.75	23.75 ± 7.64	0.42
**Vitamin D (25OHD) status**			0.25
Deficiency, < 12 ng/mL	1 (2.3)	1 (2.3)	
Insufficiency, 12–20 ng/mL	9 (20.5)	15 (34.9)	
Sufficiency, > 20 ng/mL	34 (77.3)	27 (62.8)	
**Infant**			
**Vitamin D levels**			
25OHD_2_, ng/mL	0.00 ± 0.00	0.00 ± 0.00	–
25OHD_3_, ng/mL	20.57 ± 12.66	46.01 ± 16.42	< 0.01
25OHD (total), ng/mL	20.57 ± 12.66	46.01 ± 16.42	< 0.01
**Vitamin D (25OHD) status**			< 0.01
Deficiency, < 12 ng/mL	11 (25.0)	0 (0.0)	
Insufficiency, 12–20 ng/mL	14 (31.8)	3 (7.0)	
Sufficiency, > 20 ng/mL	19 (43.2)	40 (93.0)	
**Serum bone markers**			
Calcium, mg/dL	10.60 ± 0.37	10.57 ± 0.32	0.73
Phosphorous, mg/dL	5.47 ± 0.45	5.53 ± 0.40	0.49
ALP, U/L	250.41 ± 59.71	242.79 ± 58.54	0.54
iPTH, pg/mL	19.45 (10.85–34.35)	16.3 (12.1–23.5)	0.13
**Physical growth parameters**			
Δ Weight, g	2059 ± 545	2087 ± 495	0.80
Δ Length, cm	8.5 ± 1.4	8.6 ± 1.5	0.94
Δ Head circumference, cm	4.0 ± 0.8	4.0 ± 1.0	0.70

Data are presented as mean ± standard deviation, n (%), or median (interquartile range)

Δ represents the increment in each parameter from 2 to 6 months

*25OHD*_*2*_ 25-hydroxyvitamin D_2_, *25OHD*_*3*_ 25-hydroxyvitamin D_3_, *25OHD* total 25-hydroxyvitamin D, *ALP* Alkaline phosphatase, *iPTH* Intact parathyroid hormone

The mean 25OHD concentration of all lactating women in the study was 24.43 ± 7.64 ng/mL. The maternal 25OHD concentrations were not different between the control and intervention groups (25.08 ± 7.75 vs. 23.75 ± 7.64 ng/mL, respectively; *p* = 0.42). The overall prevalence of vitamin D insufficiency and deficiency among lactating women was 27.6% (24 of 87) and 2.3% (2 of 87), respectively. There was no difference in the prevalence of vitamin D insufficiency and deficiency in lactating women between the two groups (Table 2).

Multivariate linear regression analysis, which included the study group (control or intervention), maternal 25OHD concentration, infants’ age, weight at enrollment, sex, and birth season, showed that the intervention group had vitamin D concentration 25.66 ng/mL higher than the control group [95% confidence interval (CI), 19.07–32.25); *p* < 0.00 [1]]. The model also showed the association of the maternal and infants’ 25OHD concentrations (β = 0.52; 95% CI, 0.11–0.94; *p* = 0.013) independent of whether the infants were receiving vitamin D supplementation. Other factors were not significant predictors of the infants’ 25OHD concentration. In addition, the binary regression model showed that vitamin D supplementation reduced vitamin D insufficiency and deficiency by 88.7% (relative risk, 0.11; 95% CI, 0.04–0.35; *p* < 0.01).

The associations between serum bone markers and infants’ vitamin D status were analyzed, as shown in Table 3. The iPTH concentration was 13.72 pg/mL higher in infants with a 25OHD concentration of ≤20 ng/mL than in those with a normal 25OHD concentration. No associations were found between other serum bone markers and infants’ vitamin D status.

**Table 3:** Prediction of serum bone markers at serum 25OHD concentration of ≤20 ng/mL

Serum bone markers	Beta (95% confidence interval)	*p*-value
Calcium	−0.099 (−0.255–0.056)	0.208
Phosphorous	−0.149 (−0.343–0.045)	0.129
ALP	13.899 (−13.026–40.724)	0.308
iPTH	13.724 (4.160–23.288)	0.005

*ALP* Alkaline phosphatase, *iPTH* Intact parathyroid hormone

## Discussion

This is the first study in Thailand to compare the vitamin D status of breastfed infants with and without vitamin D supplementation during the exclusive breastfeeding period. The exclusively breastfed infants were targeted in our trial because their sole source of dietary intake is breast milk, which has low in vitamin D content. In contrast, all commercially available infant formulas are vitamin D-fortified. In our study, vitamin D supplementation increased the 25OHD concentration and decreased the prevalence of vitamin D insufficiency and deficiency in breastfed infants. We found that the 25OHD concentration among unsupplemented breastfed infants was low and that fewer than half had vitamin D sufficiency. In addition, we found that approximately 30% of lactating women had serum vitamin D concentration below sufficiency level.

Limited data are available on the vitamin D status in breastfed infants in Thailand. The mean 25OHD concentration in unsupplemented breastfed infants in our study was comparable with that in breastfed infants born in summer in Greece (19.4 ± 2.8 ng/ml) [12]. The Greece study also showed a significantly higher 25OHD concentration in infants born in summer than in winter. In contrast to countries located in the northern and southern hemispheres with marked seasonal variations in weather, especially in summer and winter, Thailand has only three seasons with relatively abundant sunlight all year round. This may explain the lack of a significant association of infants’ birth season with the serum vitamin D concentration. Our study showed that unless they were receiving vitamin D supplementation, 56.8% of breastfed infants at 6 months of age had serum vitamin D concentration below sufficiency level. The prevalence of vitamin D insufficiency or deficiency among infants in tropical countries was previously expected to be low because of the large amount of sunlight in these areas. The prevalence of vitamin D insufficiency and deficiency (≤20 ng/mL) among unsupplemented breastfed infants in the present study are comparable with those reported in Hong Kong (60%) [13], but lower than those reported in India (90%) [14], Taiwan (86.1%) [15], Qatar (83%) [16], and Japan (76.9%) [17] but higher than those in Boston, MA, USA (40%) [18], Kenya (23.4%) [19], and Indonesia (16.7%) [20]. The variation in these reported prevalences was likely caused by multiple factors, such as the infants’ age, ethnicity, geographical location, and study methodology. Interestingly, an Indonesian study revealed much lower prevalences of vitamin D insufficiency and deficiency than in the present study despite the fact that Indonesia and Thailand are located at similar latitudes in the Southeast Asia region. The authors described the traditional morning sunbathing practice in the study area, which might be one of the factors that contributed to the relatively high serum vitamin D concentration among the infants in this area [20].

Our results showed that 400 IU of vitamin D supplementation daily increased the serum 25OHD concentration and contributed to an 88% reduction in the prevalence of vitamin D insufficiency and deficiency among breastfed infants. Our findings are consistent with previous studies that showed low prevalences of vitamin D deficiency and insufficiency (≤20 ng/mL) when exclusively breastfed infants were supplemented with 400 IU of vitamin D daily [21–23]. A recent systematic review determined the effect of vitamin D supplementation in breastfed infants compared with placebo [24]. The authors found six and four randomized controlled trials that determined the primary outcome of infants’ vitamin D concentration and vitamin D status, respectively. This systematic review indicated that vitamin D supplementation at 400 IU/day for breastfed infants may increase the serum 25OHD concentration and reduce the incidence of vitamin D insufficiency. However, the study was unable to confirm the benefits of vitamin D supplementation on vitamin D deficiency, the bone mineral content, the incidence of biochemical or radiological rickets, and the risk of detrimental effects in infants.

It is worth noting that no vitamin D2 (25OHD_2_) was detected in the serum of any of the infants studied. This finding was not surprising because all infants in the study were exclusively breastfed, and only vitamin D3 (cholecalciferol) was used for supplementation. Since vitamin D2 (ergocalciferol) comes from plant sources and fortified foods, this finding may assist ensure that study participants adhered to the study protocol by breastfeeding exclusively.

Serum bone markers, including iPTH and ALP, were not different between the control and intervention groups in our study. However, we found that the serum iPTH concentration was 13.7 pg/mL higher in infants with vitamin D deficiency and insufficiency (≤20 ng/mL) than in those with vitamin D sufficiency. This finding indicates the effect of vitamin D deficiency on infants’ bone health.

There is a concern regarding vitamin D toxicity while giving vitamin D supplements to infants. No infants in our study had a serum 25OHD concentration of > 100 ng/mL, the level regarded as toxicity [8]. Our study also showed that serum calcium and phosphorus were not higher in the intervention group than in the control group. This finding is consistent with a recent systematic review regarding vitamin D supplementation in breastfed infants [25].

The vitamin D concentration and prevalences of vitamin D insufficiency and deficiency among the lactating women in our study were comparable with the results of a previous study from Thailand, which reported a mean vitamin D concentration of 24.64 ± 7.72 ng/mL at delivery; concentrations of < 20 ng/mL were found in 34.0% of women [26]. Vitamin D insufficiency and deficiency in lactating women have been reported in many parts of the world. A global study showed that the mean vitamin D concentration was 28.08 ng/mL in Cincinnati, 19.44 ng/mL in Shanghai, and 19.28 ng/mL in Mexico City. Vitamin D concentrations of < 20 ng/mL were found at 4 weeks postpartum in 17, 52, and 62% of mothers in Cincinnati, Shanghai, and Mexico City, respectively [27]. The vitamin D status of lactating women should be a topic of concern because it affects the vitamin D status in breastfed infants, as shown in our study. The maternal vitamin D status during pregnancy is directly correlated with the fetal and neonatal vitamin D status, and this relationship continues during lactation [28].

To the best of our knowledge, this is the first study in Thailand to investigate the vitamin D status in exclusively breastfed infants and lactating women and to evaluate the effect of breastfed infants’ vitamin D supplementation on the vitamin D status in these infants. At the time of our study, there was no official national guideline regarding vitamin D supplementation in healthy infants. Routine vitamin D supplementation for exclusively breastfed infants has not been widely practiced by the pediatricians and general physicians who provide care for the children in the community, possibly because of lack of guidance and supporting evidence specific to the Thai population. This facilitated performance of the present randomized controlled trial, in which infants in the control group were not given vitamin D supplementation. As increasing evidence worldwide indicates the universal presence of vitamin D deficiency in exclusively breastfed infants, and once the international medical community’s authorities acknowledge the global recommendation, future studies performed in a similar fashion would likely be considered unethical.

This study has some limitations. This was an open-label study without a placebo in the control group, which could have led to bias. However, several measures were used to reduce bias, including blinding the infants’ growth assessment personals from the group allocation and targeting the objective outcome measurements. Compliance with the protocol was based solely on the parents’ interview, which could have been inaccurate. Some parents might have provided infant formula or complementary food to the infants without disclosure to the study team. The baseline vitamin D concentrations of infants and their mothers prior to entering the study were not assessed in our trial; however, the baseline characteristics of infants and their mothers in both groups were comparable. Prior to this study, routine vitamin D supplementation for exclusively breastfed infants was not practiced in our center, so none of the infants received vitamin D supplements before entering the study. Most participants in this study lived in Samut Prakan province, a city located on the outskirts of Bangkok. Therefore, the data may not represent populations in other areas of Thailand. Finally, long-term outcomes were not assessed in this study.

We hope that our findings will be utilized by healthcare authorities and policymakers to help develop a national strategy on vitamin D supplementation in Thai infants. However, because our study only included the population in Thailand’s central region and the prevalence of vitamin D deficiency in infants in other parts of the country has never been studied, the vitamin D status of infants in other areas should be investigated in order to establish national policy. Additionally, future studies demonstrating the relationship between vitamin D status and infants’ long-term health outcomes are critical to guiding the rationale for routine supplementation.

## Conclusions

Although Thailand is located in the tropical climate zone and has relatively abundant sunlight all year round, vitamin D insufficiency is not uncommon in nursing mothers and infants in Thailand. Almost one-third of lactating mothers and more than half of full-term, exclusively breastfed infants at 6 months of age have serum vitamin D concentrations below sufficiency level. However, vitamin D supplementation (400 IU/day) for these infants improves their vitamin D status and prevents vitamin D deficiency.

## Data Availability

The datasets used and/or analysed during the current study are available from the corresponding author on reasonable request.

## Abbreviations

ALP: Alkaline phosphatase
iPTH: Intact parathyroid hormone
25OHD_2_: 25-hydroxyvitamin D_2_
25OHD_3_: 25-hydroxyvitamin D_3_
25OHD: Total 25-hydroxyvitamin D
CI: Confidence interval
